# Dual release kinetics in a single dosage from core–shell hydrogel scaffolds†

**DOI:** 10.1039/c8ra05358h

**Published:** 2018-09-21

**Authors:** Finaz Khan, Debbethi Bera, Santanu Palchaudhuri, Rajesh Bera, Madhumita Mukhopadhyay, Anindita Dey, Soumyabrata Goswami, Susmita Das

**Affiliations:** Amity Institute of Applied Sciences, Department of Chemistry, Amity University Kolkata Major Arterial Road, Action Area II, Kadampukur Village, Rajarhat Newtown West Bengal 700135 India sdas@kol.amity.edu ssmtdas@gmail.com; Amity Institute of Biotechnology, Amity University Kolkata Major Arterial Road, Action Area II, Kadampukur Village, Rajarhat Newtown West Bengal 700135 India; Indian Association for the Cultivation of Sciences 2A & 2B Raja S C Mullick Road, Poddar Nagar, Jadavpur Kolkata West Bengal 700032 India; Department of Physics, Jadavpur University Kolkata West Bengal 700032 India; Department of Botany, Asutosh College Kolkata West Bengal India

## Abstract

The development of drug delivery systems with microencapsulated therapeutic agents is a promising approach to the sustained and controlled delivery of various drug molecules. The incorporation of dual release kinetics to such delivery devices further adds to their applicability. Herein, novel core–shell scaffolds composed of sodium deoxycholate and trishydroxymethylaminomethane (NaDC–Tris) have been developed with the aim of delivering two different drugs with variable release rates using the same delivery vehicle. Data obtained from XRD studies, sol–gel transition temperature measurement, rheology and fluorescence studies of the core–shell systems indicate a significant alteration in the core and the shell microstructural properties in a given system as compared to the pure hydrogels of identical compositions. The release of the model drugs Fluorescein (FL) and Rhodamine B (RhB) from the shell and the core, respectively, of the two core–shell designs studied exhibited distinctly different release kinetics. In the 25@250 core–shell system, 100% release of FL from the shell and 19% release of RhB from the core was observed within the first 5 hours, while 24.5 hours was required for the complete release of RhB from the core. For the 100@250 system, similar behaviour was observed with varied release rates and a sigmoidal increase in the core release rate upon disappearance from the shell. Cell viability studies suggested the minimal toxicity of the developed delivery vehicles towards NMuMG and WI-38 cells in the concentration range investigated. The reported core–shell systems composed of a single low molecular weight gelator with dual release kinetics may be designed as per the desired application for the consecutive release of therapeutic agents as required, as well as combination therapy commonly used to treat diseases such as diabetes and cancer.

## Abstract 2

A single LMW gelator based core–shell hydrogel with dual release kinetics.

## Graphical abstract

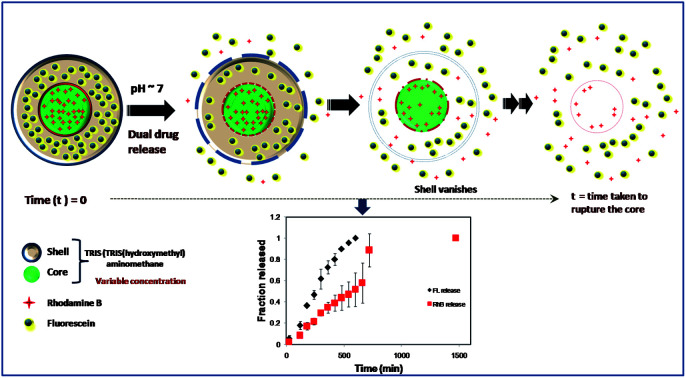

## Introduction

Drug delivery systems with controlled release characteristics enable the sustained release of bioactive materials with the desired release rate, prolonged release times and increased bioavailability.^1–5^ Hydrogels represent a class of soft materials that are of particular interest for drug delivery due to their porous structure and capability to protect the drug from hostile environmental conditions.^1,2,4^ Researchers have recently been attracted towards engineering hydrogels with organized structures for advanced biomedical applications.^6–10^ The design of core–shell hydrogel scaffolds could enable the delivery of two or more drugs with variable release kinetics through a single dosage administration. In such systems, some drugs would exhibit much faster release followed by a slow and sustained release of the other drugs from different layers of the hydrogel design. Several studies have reported the fabrication of dual delivery systems for the delivery of drugs, proteins, growth factors, and so on, based on hydrogels.^11–13^ Dual delivery systems are of extreme significance for growth factor delivery in order to drive the tissue development to completion.^14^ Mooney and co-workers developed a polymer scaffold from poly(lactide-*co*-glycolide) (PLG) that exhibited the dual delivery of growth factors VEGF and PDGF that can direct the formation of a mature vasculature, as compared to the delivery of VEGF or PDGF alone or simultaneously.^14^ A separate study reported a soy protein isolate-carbopol-polyacrylamide-based hydrogel for the combined release of two antimalarial drugs, where the hydrogel demonstrated the potential to release both the drugs for different periods of time, thereby dealing with problems like drug resistance.^15^ A core–shell composite scaffold for mimicking native skeletal muscle structure with aligned nanofiber yarn as the core and photocurable hydrogels as the shell composed of poly(carprolactone), silk fibroin, and polyaniline was reported by Wang *et al.*^11^ Jo *et al.* reported the development of polymeric core-sheath nanofibres containing colloidal arrays in the core for multi-agent delivery.^12^ Core-sheath hydrogels are also known for their application in efficient bone–tissue engineering.^13,15,16^

The studies on core–shell systems, thus far, reveal the use of primarily polymeric hydrogel systems and have rarely explored the low molecular weight gelators (LMWGs) for core–shell or multilayered designs. Low molecular weight gelator (LMWG)-based hydrogels, being mostly physical gels, afford the fabrication of core–shell systems by employing a facile approach and tuning the properties of individual layers as required for specific applications.^17^ The formation of LMWGs is mainly dictated by supramolecular assemblies formed through a combination of intermolecular H-bonding and other physical interactions such as hydrophobic forces, van der Waals forces and π–π interactions.^17^ In addition, LMWG-based systems are easily biodegradable as compared to their polymeric counterparts.

In this study, we report the fabrication of a core–shell hydrogel scaffold based on a single low molecular weight gelator, sodium deoxycholate (NaDC). For sodium deoxycholate, gelation takes place below pH 6.8, which is the p*K*_a_ of this molecule.^18^ Below this pH, the carboxylate group of NaDC is protonated and leads to increased intermolecular hydrogen bonding interactions in addition to the hydrophobic interactions between the steroid backbones of the molecules. These enhanced intermolecular interactions result in the immobilization of the buffer and the formation of hydrogels.^18^ Das *et al.*^19^ demonstrated that varying the concentration of Tris significantly modifies the sodium deoxycholate hydrogel microstructure, changing it from amorphous to highly crystalline, and fibrous to spherulites, and the various modifications have demonstrated variable drug release profiles and nano-templating characteristics.^19,20^ The Tris molecules are believed to enhance the hydrogen bonding interactions within the NaDC hydrogels as they act as bridges between the NaDC molecules, thereby increasing the size of the hydrophilic pockets within the gel network.^19^ This has been evidenced by the larger size of re-precipitated nanoparticles formed within the hydrophilic NaDC hydrogel pockets.^19^ Inspired by these studies^19,20^ and using the concept of core–shell hydrogels, in the present study we developed two core–shell hydrogel systems based on sodium deoxycholate (NaDC); the hydrogen bonding interactions were further enhanced with increasing Tris concentration. These gels were developed with the aim of obtaining dual release kinetics of two encapsulated drugs, one in the core and other in the shell, where both the core and the shell are composed of the same material with identical gelator concentration and variable amounts of gel modifier, thereby varying their properties. The core–shell scaffolds were examined for their dual and multiple release kinetics of two different model drugs. The fabricated hydrogels may demonstrate vast applicability in combination therapy, commonly used to treat cancer, or as theranostic devices that are capable of simultaneous diagnosis and therapy. Such systems may also be investigated for tissue engineering and cell encapsulation/transplantation due to the ease of the wide variation in the mechanical and microstructural properties.

## Experimental section

### Materials

Tris (tris(hydroxymethyl)aminomethane), ethanol, sodium deoxycholate (NaDC), 1-anilino-8-naphthalene sulfonate (ANS), monosodium phosphate monohydrate and disodium phosphate heptahydrate, fluorescein and rhodamine B were purchased from Sigma Aldrich and used as received.

### Preparation of hydrogels

#### Pure gels

**Scheme 1:**
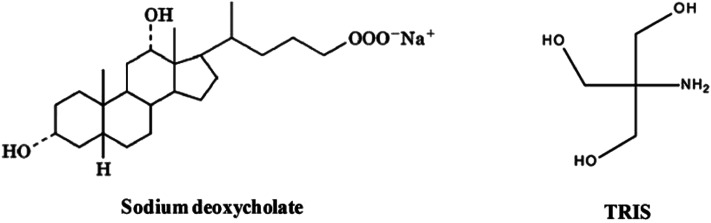
Molecular structure of NaDC and Tris.

Tris-buffer solutions of 25, 100 and 250 mM were prepared in deionized (DI) water. The pH of all the buffers was adjusted to 6 by use of concentrated and/or diluted hydrochloric acid. The pH value of 6 is ideal for the gelation of NaDC when most of the COO^−^ groups are protonated (p*K*_a_ ∼ 6.8), thereby leading to increased hydrogen bonding interactions and thus gelation (Scheme 1). The requisite amount of solid NaDC was then added to each buffer solution in the presence of a model drug and the gel was then left undisturbed for 10–15 min to set. The gelator concentration was maintained at 20 mM; Rhodamine B (RhB) and Fluorescein (FL) were used as model drugs.

#### Core–shell hydrogels

**Scheme 2:**
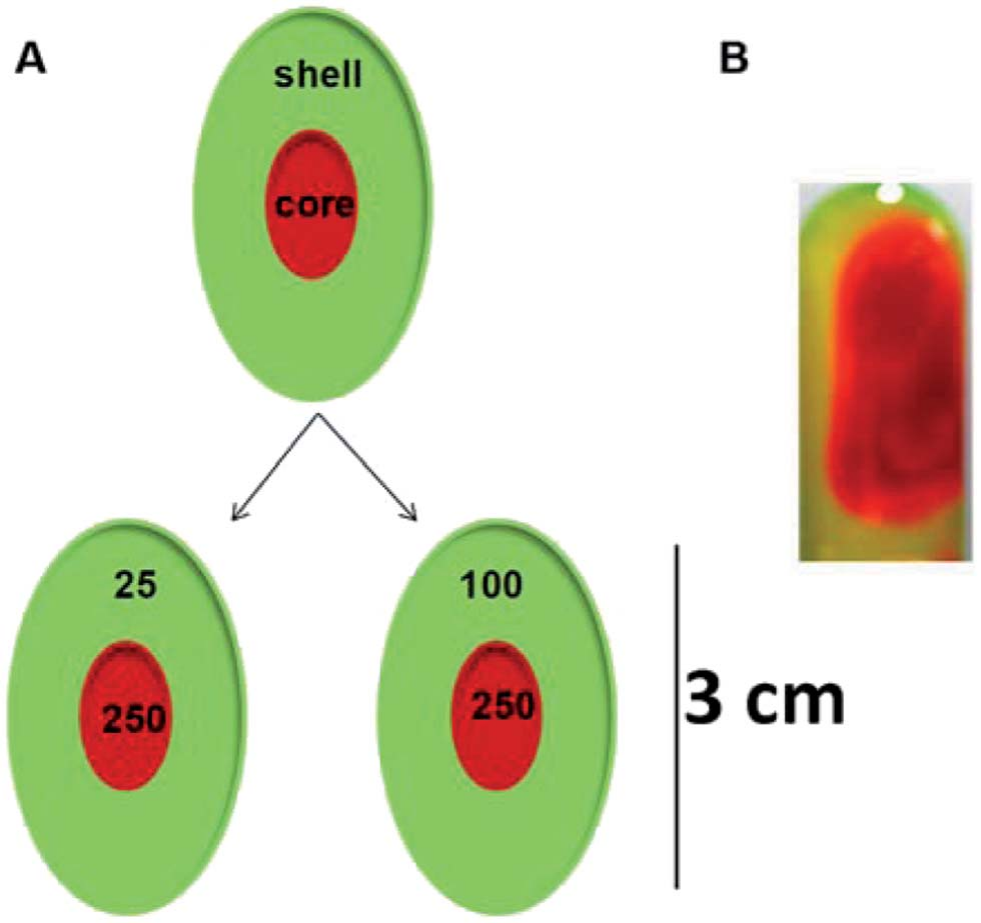
(A) Schematic representation and (B) a representative photograph of the core–shell hydrogels.

Core–shell hydrogels were prepared by first preparing 2 mL of the gel constituting the core with 250 mM Tris buffer at pH 6 containing a model drug and 20 mM NaDC. The shell, having a different Tris concentration (25 mM or 100 mM) and the same NaDC concentration, was then allowed to set around the core, possessing three times the volume of the core. A different model drug was encapsulated in the shell as compared to the core. FL was used as a model drug in the shell and RhB in the core for both the core–shell hydrogels designed and investigated in this study. The following scheme is a representative picture of the core–shell hydrogels (Scheme 2).

### Selection of the concentrations for the core and the shell

The concentration of Tris in the shell was varied from 25–100 mM while the concentration of the Tris in the core was maintained at 250 mM for both systems. The system with 25 mM Tris in the shell and 250 mM Tris in the core is represented as the 25@250 core–shell system, while the system with the 100 mM shell and 250 mM core is referred to as the 100@250 core–shell system in this study. In a previous study,^19^ it was reported that the rigidity and viscosity of the NaDC gels were higher at higher Tris concentration. As a result, in contact with aqueous media, the gel with higher Tris concentration was found to remain intact for a longer period in comparison with those having lower Tris concentration. Thus, in both systems in this study, the shell concentration was maintained lower than that of the core with the idea of developing a system with fast initial release from the shell and a slow sustained release from the core. In both cases, the shell was composed of a hydrogel with lower sol–gel transition temperature compared to the core that may also exhibit temperature-responsive drug release behaviour.

### Measurement of the sol–gel transition temperature

For measurement of the sol–gel transition temperatures, the gels were placed in a water bath with a magnetic stirring bar for homogeneous heating, and the temperature change was monitored with a thermometer. The entire system was then heated on a heating mantle with continuous stirring and the approximate phase change temperatures of the different layers were noted using the thermometer.

### X-ray analysis

X-ray diffraction patterns of the hydrogels were obtained on a Seifert 3000P X-ray diffractometer using CuKα radiation. The scanning speed was 2° per minute in the range from 5–70°. Temperature-dependent X-ray diffraction studies were performed on a PANalytical Empyrean XRD instrument using CuKα radiation.

### SEM analysis

The hydrogels were characterized by field emission scanning electron microscopy (FE-SEM, JEOL, JSM-6700F). The SEM images were obtained from samples prepared by drop casting solid film on a glass substrate.

### Rheology studies

Rheology studies of the core–shell hydrogels were performed using a rotational rheometer, Rheolab QC, Anton Paar, based on state-of-the-art technologies. For these measurements, a variable shear rate between 2–200 s^−1^ was applied on the hydrogels and their change in viscosity with increasing the shear rate was monitored at 25 °C.

### Absorption and fluorescence measurements

Absorbance measurements were performed using a Hitachi UV-Vis scanning spectrometer and the measurements were performed against an identical cell filled with buffer as the blank. Fluorescence measurements were performed using a Fluoromax-P (Horiba Jobin Yvon, NJ) fluorimeter. A 1 cm path length quartz cuvette was used to collect the fluorescence spectra. Fluorescence studies were all performed adopting a synchronous scan protocol with right angle geometry and an excitation/emission slit width of 3/3.

### Drug release study

For drug release studies, the gels were placed against a 100 mL phosphate buffer at pH 7.4, physiological pH, and the *in vitro* release of the encapsulated drugs was examined at room temperature (25 °C). The supernatant buffer was pipetted from time to time and the absorbance values at 450 and 554 nm were measured to follow the release of the two model drugs, *viz.* FL and RhB, which have been reportedly used in various studies.^21,22^ The sample was replaced in the system after each measurement was complete. The wavelengths were selected based on the fact that at 450 nm, RhB has negligible absorbance (Fig. S1†) while the absorbance of FL is appreciable. The buffer was replaced every 3 h until the gel disappeared completely.

### Cell toxicity assay/MTT assay using NMuMG Cells

NMuMG cells were left untreated or treated with 0.25 mg mL^−1^, 0.125 mg mL^−1^ and 0.0625 mg mL^−1^ of NaDC with different buffer systems or equivalent volumes of different buffer systems for 24 h. MTT (5 mg mL^−1^ in PBS) was then added at 10% of the culture media volume and incubated for 3 h at 37 °C. The medium was then removed and DMSO was added and mixed/incubated for 15 minutes. Absorbance was then measured at 540 nm.

### Cell toxicity assay/MTT assay using WI-38 cells

#### Cell lines and cell culture

Normal human lung fibroblasts, WI-38 cells, were obtained from the National Center for Cell Science (NCCS) Pune, India. The cells were cultured in respective DMEM/RPMI 1640 with 10% FBS (Fetal Bovine Serum), penicillin/streptomycin (100 units per mL), amphotericin-B (anti-fungal) at 5% CO_2_. All the treatments were done with the LD_50_ dose at 37 °C and at a cell density allowing exponential growth.

#### Cytocompatibility study using MTT assay

The cell survivability of the NaDC–Tris gels was studied in WI-38 cells using the MTT assay, following the reported procedure.^23,24^ The cells were cultured in 96-well plates at 1 × 10^4^ cells per well and exposed to various concentrations of particles (*viz.* 0 g mL^−1^, 0.03 mg mL^−1^, 0.06 mg mL^−1^, 0.125 mg mL^−1^ and 0.25 mg mL^−1^ with respect to NaDC concentrations) for 24 h.

After achieving growth, the cells were washed with 1× PBS twice, and MTT solution (450 μg mL^−1^) was added and the mixture was incubated at 37 °C for 3–4 h. The resulting formazan crystals were dissolved in an MTT solubilization buffer. Finally, the absorbance was measured by using a spectrophotometer (BioTek) at 570 nm and the value was compared with the control cells.

## Results and discussion

### Sol–gel transition temperatures of the core–shell hydrogels

The fabricated core–shell hydrogel systems were examined for their sol–gel (*T*_sg_) transition temperatures using the set up discussed in the Experimental section and the values were compared with the corresponding pure hydrogel systems. The core and shell in each of the core–shell hydrogel systems were found to undergo the sol to gel transition at distinctly different temperatures. The *T*_sg_s suggest that the formation of the core–shell system changes the intermolecular interactions and thereby the microstructural environment of the hydrogels, leading to a variation in their transition temperatures in comparison to the pure gels. A significant increase in the *T*_sg_s was observed for the core and the shell individually for both 25@250 and 100@250 core–shell hydrogel systems in comparison to the pure gels (Tables 1 & 2). Turbidity was noticed during the sol–gel transition of some of the pure gels as well as the core and the shell of core–shell systems, which indicates the phase transition of the core and shell distinctly from one another (Fig. S2†). The turbidity observed during the gel to sol transition for hydrogels with higher Tris concentration suggests the formation of larger micellar aggregates, which provide greater intermolecular hydrogen bonding bridging interactions between the NaDC molecules.^19^ The altered transition temperatures suggest an alteration of the crystallinity as well as microstructural properties of the hydrogels due to the probable modification of the supramolecular assemblies in the core–shell systems.^19,20^ These results were further investigated by XRD and fluorescence measurements.

**Table 1:** Sol–gel transition temperatures (*T*_sg_) of pure NaDC hydrogels

System	*T*_sg_ (°C)	Turbidity
25	45	No
100	67	Yes
250	65	Yes

**Table 2:** Sol–gel transition temperatures of NaDC-based core–shell hydrogels

Systems	*T*_sg_'s of shell (°C)	*T*_sg_'s of core (°C)	Turbidity
25@250	59	73	Core = yes
			Shell = no
100@250	70	74	Core = yes
			Shell = yes

### X-ray diffraction studies

**Fig. 1:**
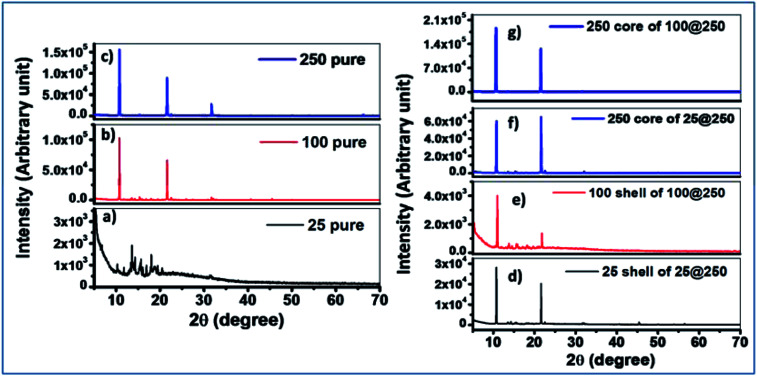
XRD patterns of the pure gel as well as the core and the shell of the core–shell xerogels. (a) 25 pure gel, (b) 100 pure gel, (c) 250 pure gel, (d) 25 shell of 25@250 gel, (e) 100 shell of 100@250 gel, (f) 250 core of 25@250 gel, (g) 250 core of 100@250 gel.

X-ray diffraction results of pure individual gels with the same composition as the cores and the shells were compared with the core–shell hydrogels. The 25 mM pure hydrogel exhibited a diffraction spectrum with a relatively low signal to noise ratio, suggesting less crystalline and more amorphous characteristics of the hydrogel. The 100 mM pure gel demonstrated two distinct diffraction peaks at 2*θ* values of 10.8° and 21.6°, respectively whereas the 250 mM pure gel exhibited three distinct peaks at 2*θ* values of 10.8°, 21.6°, and 31.7°. This difference in the microstructural packing of the pure 250 mM hydrogel accounts for the deviation in the observed trend of sol–gel transition temperature where, in spite of the increase in Tris concentration, no significant increase in the sol–gel transition temperature was observed for the 250 mM hydrogel in comparison to the 100 mM pure gel. XRD studies suggest a considerable alteration in the 250 mM hydrogel constituting the core, as compared to the pure 250 mM gel for both the 25@250 and 100@250 core–shell systems where three diffraction peaks were observed for the pure gel, while the 250 mM core in both the core–shell systems showed only two peaks at 2*θ* values of 10.8° and 21.6°. The third peak at 31.7° disappeared from the spectrum of the 250 mM core of both the core–shell hydrogel systems. In addition, the diffraction intensities were found to be reduced upon formation of the core with 250 mM Tris in comparison to pure the 250 mM hydrogel. This suggests that the formation of the core–shell structure of hydrogels results in an altered hydrogel microstructural assembly along with the variation in its crystallinity. The diffraction peak positions of the 25 mM and 100 mM shells in both the core–shell systems were similar to those of the respective pure gels. However, the relative diffraction peak intensities at 2*θ* values of 10.8° and 21.6° of the shells were found to be significantly different from the pure hydrogels, indicating their altered supramolecular assemblies (Fig. 1). These variations in the XRD results of the pure hydrogels with variable Tris concentrations as well as the pure hydrogels compared to the same composition of the core or shell of core–shell hydrogels are attributed to the altered hydrogen bonding and hydrophobic interactions. The influence of hydrogen bonding on crystallinity has been investigated and reported in several studies.^25^ The effect of altered hydrogen bonding is also reflected in the sol–gel transition temperatures of the hydrogels (Tables 1 & 2). The implied alteration in hydrogel microstructure due to the formation of the core–shell systems was further investigated using fluorescence spectroscopy.

XRD measurements were also performed at high temperatures for two gels to understand the phase changes of the gels with temperatures. Temperature-dependent XRD studies at 50 and 70 °C for both 25 and 250 mM gels of the 25@250 core–shell system demonstrated some additional peaks at higher *θ* values, namely at 30, 45 and 50° for the 25 mM gel and at 30, 45, 50 and 55° for the 250 mM gels (Fig. S3†). The appearance of peaks at higher *θ* values indicate some microstructural changes at higher temperatures, which are expected at 50 and 70 °C since these temperatures lie close to the sol–gel transition temperatures of the 25 mM and 250 mM, respectively, where the hydrogen bonding interactions within the gels are drastically altered. However, the XRD measurements were carried out on air-dried gels and thus transformation to the sol state is not possible under experimental conditions.

### Scanning electron microscopy

**Fig. 2:**
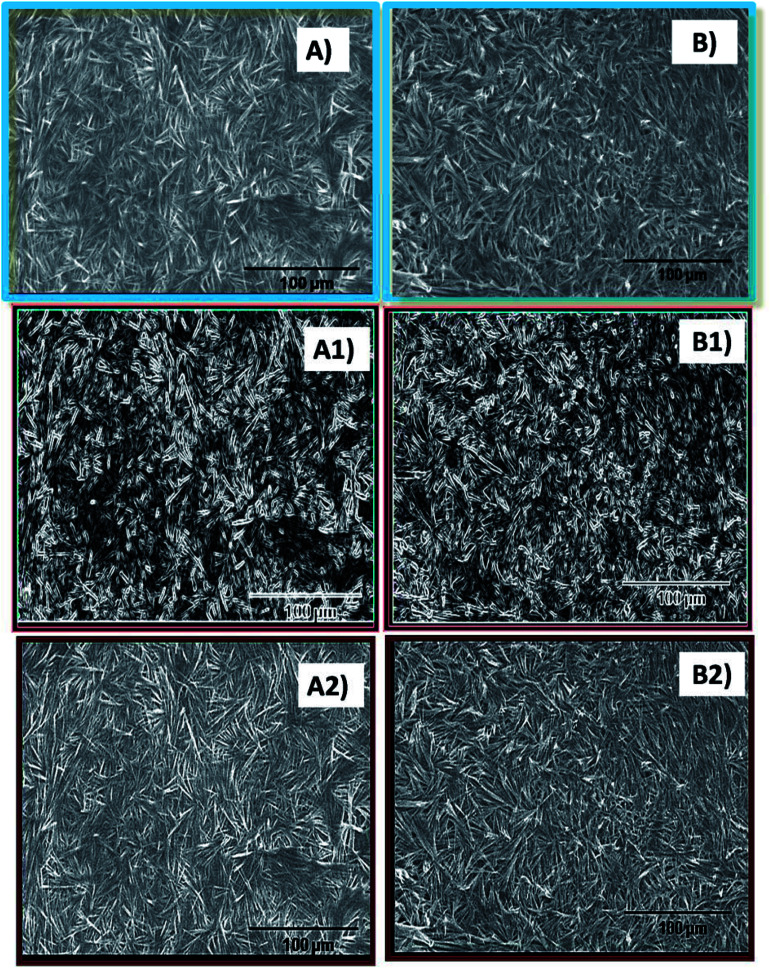
Scanning electron micrographs of (A) the 100 shell and (B) the 250 core of the 100@250 core–shell hydrogel. (A1), (A2), (B1) and (B2) represent the defined edges and sharpened images of (A) and (B), respectively, as obtained using ImageJ.

Scanning electron microscopy studies were performed for the cores and shells of the core–shell hydrogel systems. The micrographs indicate the presence of fibrous structures in the 250 mM core as well as the 25 and 100 mM shell of the hydrogel systems (Fig. 2). Due to the higher density of the gel fibers as observed in the scanning electron micrographs, other images with sharpened and defined edges were generated for a better morphological understanding and these images clearly indicate the presence of fibres in the hydrogel scaffolds. This particular structural feature is similar to the morphological features of the pure NaDC hydrogels of identical concentrations as observed in the literature.^19,20^ Thus, the formation of core–shell hydrogels do not affect the gross morphological structure of deoxycholate hydrogels, although indications of alterations in the hydrogel microstructural organization are evident from the XRD data.

### Rheological study of the core–shell hydrogels

**Fig. 3:**
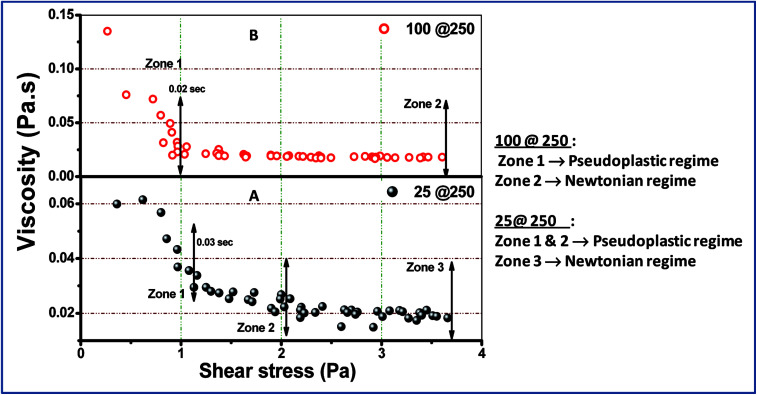
Shear stress-dependent viscosity change in (A) 25@250 and (B) 100@250 core–shell hydrogels.

**Fig. 4:**
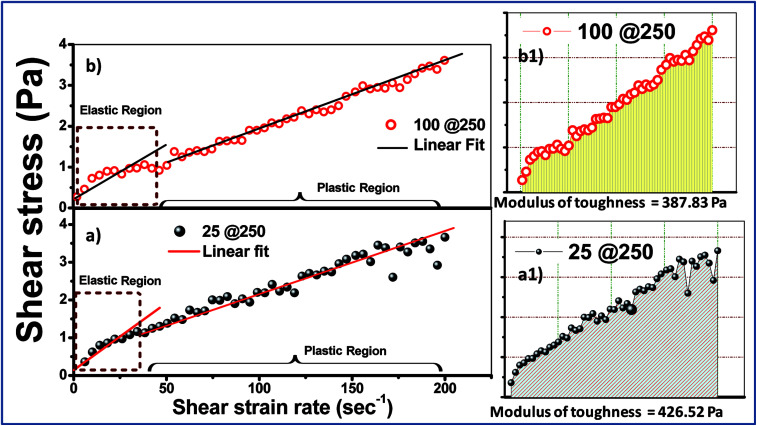
Shear stress *vs.* shear strain rate plots of the (a) 25@250 and (b) 100@250 hydrogels. (a1) and (b1) represent the modulus of toughness for the 25@250 and 100@250 core–shell hydrogels, respectively.

Examination of the rheology study (Fig. 3 and 4) allowed the understanding of the melt flow and mechanical properties of the core–shell hydrogels and thus the evaluation of their potential application in drug delivery. Fig. 3 shows the viscosity behaviour of the developed core–shell hydrogels in response to the applied shear stress up to 4 Pa. At higher stress (more than 4 Pa), the viscosity was found to be unaltered and was not reported in the present study. Both 25@250 and 100@ 250 gels exhibit non-Newtonian shear thinning, wherein the viscous drag tend to decrease asymptotically.^26^ Hydrogels that exhibit shear thinning upon the application of a shear stress are highly desirable for injectable therapeutic delivery vehicles.^27^ For the 25@250 core–shell hydrogel, the pseudoplastic shear thinning nature is found to exhibit two zones, *viz.* zone 1 and 2 (Fig. 3A), corresponding to the sharp and shallow reduction of viscosity. However, the 100@250 hydrogel follows a steep lowering of viscosity till 1 Pa. Shear thinning of the core–shell hydrogels with increasing shear rate/stress has wide applicability in injectable drug delivery vehicles. In the study by Das *et al.*,^19,20^ it was observed that the loss modulus which is equivalent to viscosity of pure 25 mM and 100 mM NaDC gels was found to gradually increase with the initial increase in the shear frequency, followed by a frequency independent region.^19^ The study also revealed shear thickening for 250 mM hydrogels. However, the rheological properties of both the 25@250 and 100@250 core–shell hydrogels were significantly different from the pure gel. Ideal Newtonian viscosity (shear independent behaviour) was observed after 2 Pa for the 25@250 and 1 Pa for the 100@250 hydrogel. This signifies that the 25@250 gel is able to withstand the viscoelastic thinning behaviour up to higher shear stress, as compared to the 100@250 gel. This observation complements the XRD data where a significant increase in crystallinity was observed in the 25 shell of the 25@250 hydrogel as compared to the pure 25 mM hydrogel. In contrast, the diffraction intensity of the 100 shell of the 100@250 hydrogel was found to be less than the pure 100 mM gel as well as the 25 shell of the 25@250 hydrogel. This indicates lower shell crystallinity and thus lowered resistance to the viscoelastic thinning of the 100@250 core–shell systems.

The mechanical behaviour of the formed gels can be more clearly studied using the stress *vs.* strain plot shown in Fig. 4. Owing to the fact that the experimental gels are pseudoplastic in nature, the stress *vs.* shear strain plot shows two different regions, *viz.* elastic and plastic, which can be best fitted using two straight lines.^28^ The area under the stress–strain curve up to the elastic limit depicts the modulus of resilience, which signifies the ability of the material to store or absorb energy without permanent deformation. The more significant factor is the modulus of toughness, which is represented in Fig. 4a1 and b1 and is calculated from the area under the stress *vs.* strain curve. The toughness modulus is a measure of the impact loading that a structure can withstand before failure. It was observed that the 25@250 gels have a wider pseudoplastic regime (Fig. 3A), and thus a higher modulus of toughness (∼427 Pa). According to first order principles, the toughness modulus estimates the measure of the external force required to disintegrate the gel properties. During practical application as a drug carrier, the applied force is in the form of a variable chemical environment present *in vitro*, wherein the intracellular fluid does not exhibit static loading. Thus, it is important to have an understanding of the toughness modulus of the gels that are designed as drug delivery vehicles.

### Analysis of hydrogel microstructure using fluorescence spectroscopy

The perception of the hydrogel microstructural modification in core–shell systems in comparison to the pure gels as well as microstructural differences of the core relative to the shell in each hydrogel system is essential for estimating the possibility of their exhibiting dual release kinetics. This investigation was performed using ANS [8-anilinonaphthalene-1-sulfonic acid] as the fluorescent hydrophobicity probe. Both the core and the shell of the core–shell systems were prepared with ANS and their fluorescence emissions were examined.

In pure gels, increasing Tris concentration caused a gradual blue shift in the ANS emission from 520 nm in the pure buffer to 440 nm in the NaDC gels with the highest concentration of Tris (250 nm) (Fig. S4†). This blue shift indicates that ANS is bound to a more hydrophobic environment that is caused by the enhanced networking at higher Tris concentration.^19^ However, the initial increase was followed by a drop in ANS emission intensity in gels at higher Tris concentration, which indicated the decreased molecular rigidity in these hydrogels.^29,30^ It is well explained in literature that NaDC hydrogels with higher Tris concentration contain larger aqueous pools due to the bridging of Tris molecules between the deoxycholate molecules through hydrogen bonding interactions.^19^

**Fig. 5:**
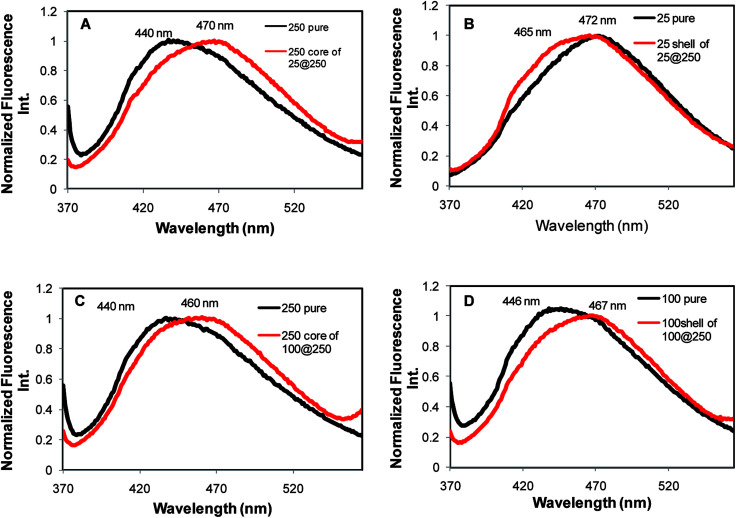
Fluorescence emission of the hydrophobic probe ANS in the pure hydrogel as well as the core and shell with the corresponding composition in core–shell hydrogels.

Examination of the ANS emission in core–shell systems yielded interesting observations. ANS in the 25 mM shell of the 25@250 core–shell system demonstrated an emission at 465 nm, which was blue-shifted by 7 nm with respect to the pure 25 mM gel, while the 250 mM core demonstrated an ANS emission at 470 nm, which is red shifted by 30 nm in comparison to the pure 250 mM gel (Fig. 5). The ANS emission intensity in the shell was considerably higher than that in the core (data not shown). The data indicate that in the 25@250 gel, ANS is present in a relatively more hydrophobic environment in the shell with the restricted mobility of the chromophoric unit in comparison to the core as well as to the pure 25 mM gels. In contrast, in the 100@250 mM core–shell system, the 250 mM core is more hydrophobic (ANS emission at 460 nm) compared to the 100 mM shell (ANS emission at 467 nm) (Fig. 5C). Both the core and the shell of the 100@250 core–shell hydrogel exhibited a red-shifted ANS emission with respect to the corresponding pure gels. This is suggestive of a relatively less hydrophobic microenvironment in the core–shell system in comparison to the pure gels. Altogether, the fluorescence measurements point towards a significant micro-environmental difference between the core and the shell with respect to the pure gels as well as the core and the shell of the individual core–shell hydrogels. These observations in conjunction with XRD and melting point measurements indicate, to some extent, an expected difference in release kinetics from the core with respect to the shell of such systems, due to the observed alterations in the gel microenvironment. The results also suggest the ease of formation of a new core–shell system by variation of the Tris concentration in the shell, thereby proposing the tunability in the hydrogel design as per the desired application.

### Drug release from core–shell hydrogels

**Fig. 6:**
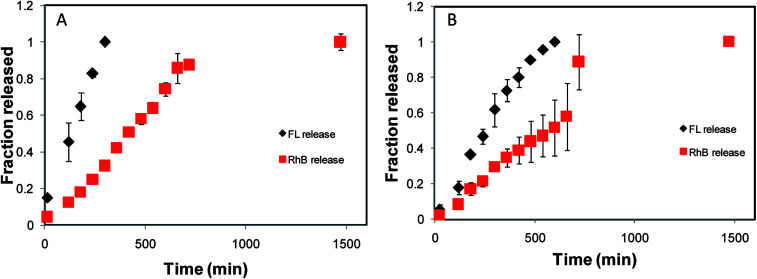
Drug release profiles of (A) 25@250 and (B) 100@250 core–shell hydrogels. The black diamonds represent the release of FL from the shell and the red squares represent the release of RhB from the core. The standard deviations are obtained for the studies performed in triplicates.

The release of model drugs FL and RhB from the shell and the core, respectively, was followed using UV-visible absorption measurements at 450 nm for FL and 554 nm for RhB. Fig. 6A, representing the release profile of a 25@250 core–shell system, suggests two distinct rates of release from the core and the shell. The fraction of FL released from the 25 mM shell was found to linearly increase with time and nearly 100% release was observed in 300 minutes when the shell was found to disappear. In contrast, only 32% of RhB was released during the same time span from the core. The core demonstrated linear release kinetics up to 660 min when 85% of RhB was released. It is also interesting to note that 24.5 h (1470 min) were required for 100% release from the core, whereas the release from the shell was complete in the initial 5 h (300 min) period. Thus, the core–shell hydrogel was proved to demonstrate dual release kinetics of two different drugs: one encapsulated in the shell for fast release and the other in the core for prolonged and sustained release. In order to establish the hydrogel design and to prove that the observed release kinetics is a property of the core–shell design and not the characteristic of the model drug, reverse encapsulation was performed where RhB was encapsulated in the shell of the core–shell design and FL was encapsulated in the core. The release of the model drugs from the core and the shell was examined and the release profile exhibited distinct release kinetics from the core and the shell, as expected. From the 25@250 hydrogel with reverse encapsulation, 100% release was complete from the shell in about 360 min, while only 19% release was observed from the core during the same time span. Thus, it may be concluded that the dual release kinetics of the designed delivery vehicles are primarily dominated by the core–shell design; however, the release percent may vary with nature of the encapsulated drug.

In the 100@250 core–shell system, the shell demonstrated 100% release in 600 min, while in the same time span the release from the core was 51%. From the core, 100% release was observed in 1470 min (Fig. 6B). In all the core–shell systems, the release from the core was accelerated after the shell completely disappeared, signifying that the shell acts as a protective layer and thus controls the release kinetics of the core. Complete release time from the shell or, in other words, the shell degradation time of the 25@250 core–shell system was found to be nearly half of the time required for the 100@250 hydrogel. This observation contradicts the measure of toughness modulus as recorded in the rheology studies and the greater stability of the shell in the 100@250 gel may be attributed to greater hydrogen bonding in the presence of higher Tris concentration as compared to the 25@250 gel. In addition, it should also be noted that in the *in vitro* drug release studies, the inherent release kinetics of the hydrogel design was investigated in the absence of any applied stress. Thus, the drug release study indicates that the physical properties and composition of the gel constituting the shell governs the release characteristics of the core–shell systems investigated, consequently allowing the design of systems with tunable release properties.

### Cell viability studies

**Fig. 7:**
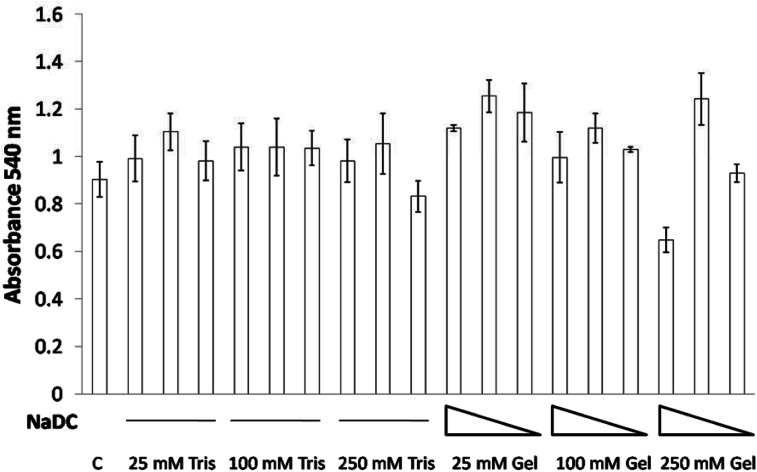
The effect of the NaDC–Tris hydrogel on NMuMG cell viability. Cells were treated with different Tris buffer systems and NaDC–Tris hydrogels (see Material and methods) for 24 h. Toxicity/cell viability was measured by MTT assay. Data are shown as mean ± SD. C represents the control/untreated cells.

Cell viability studies on 0.25 mg mL^−1^, 0.125 mg mL^−1^ and 0.0625 mg mL^−1^ of 25, 100 and 250 mM NaDC–Tris hydrogels as well as proportionate amounts of 25, 100 and 250 mM Tris, pH 6 were performed with mouse breast epithelial cells (NMuMG). The study revealed that even high concentrations of 0.25 mg mL^−1^ of the hydrogel in combination with 25 mM or 100 mM Tris at pH 6 failed to exhibit any toxic effects on NMuMG cells when incubated for 24 h (Fig. 7). Only when cells were treated with 0.25 mg mL^−1^ of the hydrogel in combination with 250 mM Tris at pH 6 did we see cell death in a small percentage of the population. Even then, at hydrogel concentrations of 0.125 or 0.0625 mg mL^−1^, no toxicity was found (Fig. 7) The studies with the corresponding buffers, 25–250 mM Tris at pH 6, indicated the insignificant effect of the buffer system on the cell survivability. Thus, the concentration-dependent effect observed for NaDC–Tris hydrogels was attributed to the hydrogel's inherent characteristics resulting from its microstructural differences at higher buffer concentration as discussed in an earlier section.

The core–shell hydrogel designs presented in this study are comprised of the shells constituted by 25 and 100 mM hydrogels, which were found to gradually dissolve in a total volume of 200 mL buffer over 6 h with a final NaDC concentration of 0.24 mg mL^−1^, and both 25 and 100 mM NaDC–Tris, pH 6 hydrogels demonstrated no toxicity at these concentrations (Fig. 7). The 250 mM NaDC–Tris, pH 6 hydrogel constituting the core of both 25@250 and 100@250 core–shell systems was exposed to the environment once the shell disappeared and then dissolved over 18 h in 600 mL of buffer, resulting in a final concentration of 0.026 mg mL^−1^ of NaDC. At this concentration of NaDC, the 250 mM hydrogel did not exhibit any toxic effect. Assuming that the gels will be exposed to much larger volumes of continuously circulating body fluid under real conditions, we can state based on our studies that these core–shell hydrogel based delivery vehicles will exhibit insignificant toxicity to the cells. Sodium deoxycholate coated zein nanoparticles have been reported as stable biocompatible drug delivery systems.^31^ Furthermore, NaDC and Tris, being soluble substances, are expected to possess lower circulation times in comparison to insoluble delivery vehicles and thus faster excretion from the body is expected.^32^ Thus, the core–shell hydrogel scaffold formulation using the NaDC–Tris system may be considered as appreciably biocompatible as observed from the cell viability results.

**Fig. 8:**
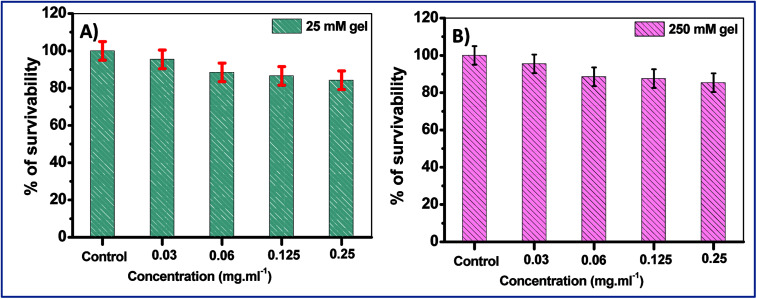
The effects of (A) 25 mM and (B) 250 mM NaDC–Tris hydrogel on WI-38 cell viability with varying concentrations of NaDC.

In order to further confirm these results, the cell survival of two of the hydrogels, namely the 25 mM and 250 mM gels, were examined with normal human lung fibroblasts, WI-38 cells. The studies revealed that for both 25 mM and 250 mM hydrogels, cell death was not very significant, with ∼85% cell viability at the maximum concentration (0.25 mg mL^−1^ of NaDC) studied for a 24 h incubation period (Fig. 8). However, the only difference observed here was in the viability of the 250 mM hydrogel at 0.25 mg mL^−1^ of NaDC concentrations, where the survival percentage was significantly higher compared to the mouse cells. It was observed that with variable NaDC concentration, the cell survival percentage was similar for both 25 and 250 mM hydrogels where 25 and 250 represent the Tris concentration that acts as the gel modifier. These results suggest that the toxicity of Tris to the cells is negligible. The results support the observations made from the studies with mouse breast epithelial cells and once again suggest the significant biocompatibility of the studied hydrogels as drug delivery vehicles.

## Conclusions

In summary, this work presents the fabrication of core–shell hydrogel systems composed of the same LMW gelator NaDC, possessing identical concentrations of the gelator and variable concentration of Tris in the core and the shell. The factor that creates the difference between the core and the shell is the concentration of Tris, which acts as the gel modifier. The core and the shell of the hydrogels present considerably distinct microenvironments with respect to the pure gels of identical composition as well as with respect to each other in a given core–shell system, as observed from the XRD and fluorescence results. This observed microstructural difference between the core and shell accounts for the appreciably different release kinetics exhibited by the core and shell of a given core–shell hydrogel. This system is reportedly the first of its kind that is composed of purely LMW biodegradable hydrogelators that will have an insignificant possibility of accumulation since they are soluble in aqueous medium, and thus will be excreted from the body through complete dissolution over 6–36 hours as suggested by the studies. In addition, the difference in the sol–gel transition temperature of the shell compared to the core may allow their applicability as temperature responsive delivery systems.^33^ Cell viability studies with the hydrogels suggest the insignificant toxicity of the designed hydrogels towards mouse breast epithelial cells (NMuMG) and human normal lung fibroblast WI-38 cells, thereby ascertaining their applicability as drug delivery vehicles. Thus, the hydrogel design demonstrates great potential for the delivery of two small drug molecules with complementary activity and with noticeably different release rates using a single delivery system.

## Conflicts of interest

There are no conflicts to declare.

## Supplementary Material

RA-008-C8RA05358H-s001

## References

[cit1] Du X., Zhou J., Shi J., Xu B. (2015). Chem. Rev..

[cit2] Kamaly N., Yameen B., Wu J., Farokhzad O. C. (2016). Chem. Rev..

[cit3] Das D., Pal S. (2015). RSC Adv..

[cit4] Vermonden T., Censi R., Hennink W. E. (2012). Chem. Rev..

[cit5] Esposito C. L., Kirilov P., Roullin V. G. (2018). J. Controlled Release.

[cit6] Nie J., Lu W., Ma J., Yang L., Wang Z., Qin A., Hu Q. (2015). Sci. Rep..

[cit7] Yang J., Deng L.-H., Han C.-R., Duan J.-F., Ma M.-G., Zhang X.-M., Xu F., Sun R.-C. (2013). Soft Matter.

[cit8] Sun N., Wang T., Yan X. (2017). RSC Adv..

[cit9] Xia S., Song S., Ren X., Gao G. (2017). Soft Matter.

[cit10] Bai J., Gong Z., Wang J., Wang C. (2017). RSC Adv..

[cit11] Wang L., Wu Y., Guo B., Ma P. X. (2015). ACS Nano.

[cit12] Jo E., Lee S., Kim K. T., Won Y. S., Kim H.-S., Cho E. C., Jeong U. (2009). Adv. Mater..

[cit13] Perez R. A., Kim M., Kim T.-H., Kim J.-H., Lee T. H., Park J.-H., Knowles J. C., Kim H.-W. (2014). Tissue Eng., Part A.

[cit14] Richardson T. P., Peters M. C., Ennett A. B., Mooney D. J. (2001). Nat. Biotechnol..

[cit15] Aderibigbe B. A., Mhlwatika Z. (2016). J. Appl. Polym. Sci..

[cit16] Barry J. J. A., Howard D., Shakesheff K. M., Howdle S. M., Alexander M. R. (2006). Adv. Mater..

[cit17] Panda J. J., Mishra A., Basu A., Chauhan V. S. (2008). Biomacromolecules.

[cit18] Mukhopadhyay S., Maitra U. (2004). Curr. Sci..

[cit19] Das S., deRooy S. L., Jordan A., Chandler L., Negulescu I. I., El Zahab B., Warner I. M. (2012). Langmuir.

[cit20] E McNeel K., Das S., Siraj N., I Negulescu I., M Warner I. (2015). J. Phys. Chem. B.

[cit21] Fisher K. A., Huddersman K. D., Taylor M. J. (2003). Chem.–Eur. J..

[cit22] Zhang R., Hummelga M., Lv G., Olin H. (2011). Carbon.

[cit23] Pramanik A., Laha D., Pramanik A., Chattopadhyay S., Dash S. K., Roy S., Pramanik P., Karmakar P. (2016). Mater. Sci. Eng., C.

[cit24] Laha D., Pramanik A., Chattopadhyay S., Dash S. K., Roy S., Pramanik P., Karmakar P. (2015). RSC Adv..

[cit25] McKiernan R. L., Heintz A. M., Hsu S. L., Atkins E. D. T., Penelle J., Gido S. P. (2002). Macromolecules.

[cit26] Mandal S., Chakroborty S. (2017). Phys. Rev. Fluids.

[cit27] Li J., Mooney D. J. (2016). Nat. Rev. Mater..

[cit28] Jones R. M. , Deformation Theory of Plasticity, Bull Ride Publishing, Virgina, USA, 2009

[cit29] Dykman J., Weltman J. K. (1970). J. Cell Biol..

[cit30] Alizadeh-Pasdar N., Li-Chan E. C. Y. (2000). J. Agric. Food Chem..

[cit31] Gagliardi A., Paolino D., Iannone M., Palma E., Fresta M., Cosco D. (2018). Int. J. Nanomed..

[cit32] Liu Z., Chen K., Davis C., Sherlock S., Cao Q., Chen X., Dai H. (2008). Cancer Res..

[cit33] Dai S., Ravi P., Tam K. C. (2009). Soft Matter.

