# Supplementary material for: Dual release kinetics in a single dosage from core–shell hydrogel scaffolds
Source: RSC Adv. 2018 Sep 21;8(57):32695–706. doi: 10.1039/c8ra05358h (PMC9086265; doi:10.1039/c8ra05358h)
Supplement: RA-008-C8RA05358H-s001 [file RA-008-C8RA05358H-s001.pdf]

Electronic Supplementary Material (ESI) for RSC Advances.
This journal is © The Royal Society of Chemistry 2018

Dual Release Kinetics in Single Dosage from Core-Shell Hydrogel Scaffolds

Finaz Khan,[a] Debbethi Bera,[d] Santanu Palchaudhuri,[b] Rajesh Bera,[c] Madhumita Mukhopadhyay[a], Anindita Dey,[e] Soumyabrata Goswami[a] and Susmita Das [a,*]

[a] Amity Institute of Applied Sciences, Department of Chemistry, Amity University Kolkata, Major Arterial Road, Action Area II, Kadampukur Village, Rajarhat, Newtown, West Bengal 700135, India.

[b]Amity Institute of Biotechnology, Amity University Kolkata, Major Arterial Road, Action Area II, Kadampukur Village, Rajarhat, Newtown, West Bengal 700135, India.

[c] Indian Association for the Cultivation of Sciences, 2A & 2B Raja S C Mullick Road, Poddar Nagar, Jadavpur, Kolkata, West Bengal 700032, India.

[d]Department of Physics, Jadavpur University, Kolkata, West Bengal 700032, India

[e]Department of Botany, Asutosh College, Kolkata, West Bengal, India

*Corresponding authors email id:sdas@kol.amity.edu; ssmtdas@gmail.com

Supporting Information

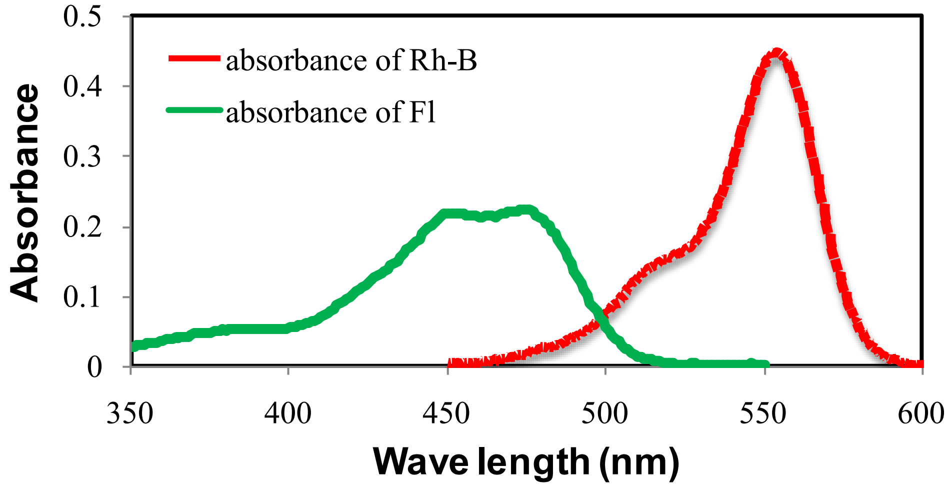

Figure S1: Absorption spectra of Fluorescein and Rhodamine B in water

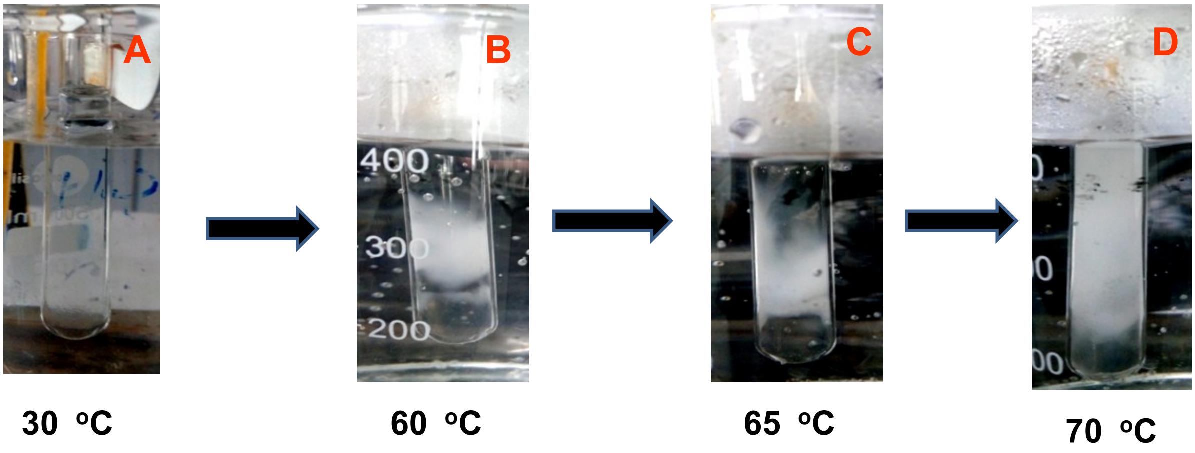

Figure S2: Sol-gel transition process of 25@250 gel showing turbidity of the core while the shell remains transparent. As the core undergoes transition to sol it slowly diffuses into the already transformed shell.

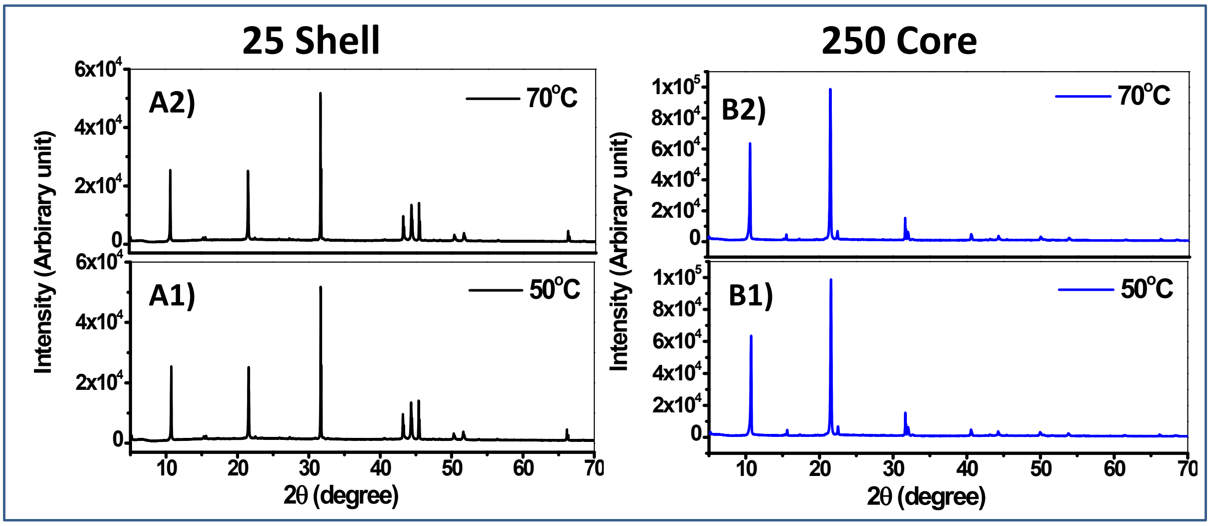

Figure S3: Temperature dependent XRD of 25 and 250 mM shell and core of 25@250 xerogels.

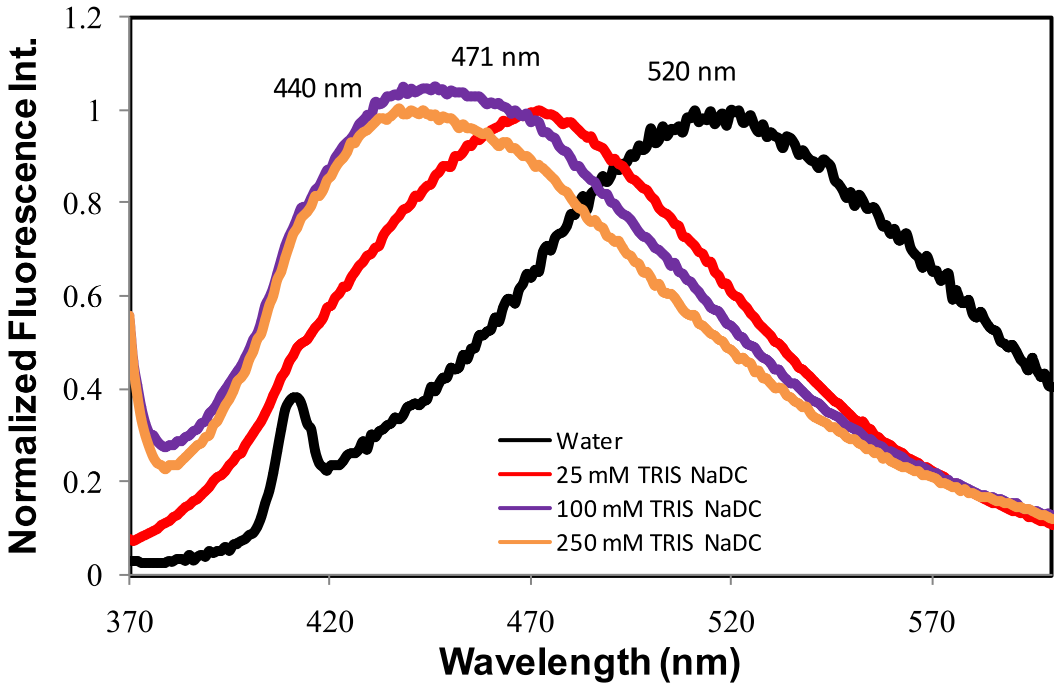

Figure S4: Fluorescence emission spectra of hydrophobicity probe ANS in the pure hydrogels with varying TRIS concentration.
